# Spinal Meningitis, or Spotted Fever

**Published:** 1866-06

**Authors:** G. C. Paoli


### CHICAGO MEDICAL JOURNAL.

Vol. XXIII. JUNE, 1866. No. 6.

### ORIGINAL CONTRIBUTIONS.

*Spinal Meningitis, or Spotted Fever.* By G. C. Paoli, M. D.

(Read before the Chicago Medical Society, May 5, 1866.)

Mi. Kadish, a girl, seven years of age, was taken suddenly sick with a chill on the 1st of April. On the 3d of April, they requested my attendance. I found my little patient suffering with the following symptoms: The head was thrown back and so firmly fixed that she could not move it without at the same time moving the body. The smallest pressure applied to the neck and back was exceedingly painful. Her appearance was that of extreme anxiety and distress. The temperature of the skin was cold, and ecchymoses, or large blue spots, were visible on both arms and lower extremities. The pulse frequent, small and compressible. Her tongue dry and covered with a yellow fur. Bowels constipated and very tender to the touch, particularly over the transverse colon. She had not slept since the commencement of her illness. There was also intense headache and great difficulty in deglutition. Vomiting had occurred several times.

From those symptoms I have described, I saw that the disease was evidently a spinal meningitis, or spotted fever, in the worst form, and I nourished a very faint expectation that my little patient would survive this formidable malady which is so much dreaded by our profession.

As I kept a journal in this case, I could have detailed for you to-night the daily changes; but to avoid tediousness, I shall permit myself only to notice the peculiarity of the symptoms and the treatment.

My first endeavor was to produce a reaction. For that purpose, I let the patient take a warm bath, which produced a gentle perspiration. After the bath I gave an injection, which produced an evacuation of hard, dark excrement. To the spine I applied flannel dipped in oil of turpentine, which produced considerable irritation of the skin.

Later in the evening, I gave her a calomel powder, which produced evacuation of the bowels of a very offensive or tarry-looking character.

It was with great difficulty that the patient could swallow anything, so that I had, for four successive days, to feed her with injections of beef-tea and milk. It was on the fifth day she commenced to drink milk, which constituted her food during her illness. The internal remedy, which I most relied on, was bromide of potassium, of which I gave 10 grs. every other hour. It seemed to have a sedative influence both as well to relieve the morbid sensitiveness as to procure sleep. Occasionally I gave fluid extract of cannabis indica, which, when the pain in the head was very intense, had a soothing effect. Besides the turpentine as an internal remedy, I used mercurial ointment and camphorated liniment.

During the progress of the disease the vital powers were very much depressed, so I prescribed musk. Although this powerful nervine is so scarce, I obtained an excellent article. The disease, which had a duration of five weeks, had its peculiar symptoms, which are worth mentioning, viz: every other day the temperature of the skin was more or less similar to what is often observed in a dull fever and ague; the tonic contraction of the muscles in the back were protracted; pains in the different articulations, like wandering rheumatic pains, which often prevented movement of her arms; and loss of hearing, from which she is now slowly recovering.

This case, as well as the previous cases that have come under my observation, convince me that an active poison is, for a long time, lingering in the system.

